# A Case of Anaphylactic Shock Due to Levofloxacin Eye Drops Confirmed by a Prick Test

**DOI:** 10.7759/cureus.53804

**Published:** 2024-02-07

**Authors:** Isamu Ikeda, Satoshi Fukushima

**Affiliations:** 1 Department of Dermatology, Omuta Tenryo Hospital, Omuta, JPN; 2 Department of Dermatology and Plastic Surgery, Faculty of Life Sciences, Kumamoto University, Kumamoto, JPN

**Keywords:** eye infections, ofloxacin, prick test, anaphylactic shock, levofloxacin

## Abstract

Topical levofloxacin has been used safely, but it can induce life-threatening hypersensitivities. We report a case of anaphylactic shock caused by levofloxacin eye drops during the treatment of a corneal injury, confirmed by a prick test. Reported cases of hypersensitivity to levofloxacin and its racemate ofloxacin eye drops are also summarized.

## Introduction

The use of levofloxacin eye drops is increasing as the number of bacteria resistant to existing antibiotics increases. Although fluoroquinolones are well-tolerated antibacterial agents, they have also been reported to cause serious side effects such as anaphylaxis [1,2]. The cross-reactivity of quinolones is discussed also [2]. We report a case of anaphylactic shock caused by levofloxacin eye drops. Cases of serious hypersensitivity reported with levofloxacin and its racemate ofloxacin are also summarized.

## Case presentation

A 65-year-old man was admitted to the emergency department complaining of eye pain, nausea, and cold sweats with hypotension of 56/42 mmHg shortly after using oxybuprocaine 0.4% and levofloxacin 1.5% eye drops at an ophthalmology clinic. The episode occurred during the removal of a corneal foreign body (wood particle). He had received the same treatment for corneal foreign body five years earlier without adverse reactions. Other than that, no history of topical or oral quinolones including levofloxacin was found. He had no history of hypersensitivity to drugs and/or foods.

On admission, his blood pressure was 118/72 mmHg but soon dropped to 101/61 mmHg with tremors. A mild rash was observed on the chest and abdomen. He recovered after an intramuscular injection of adrenaline 0.3 mg and a saline bolus. The rash has also disappeared. Methylprednisolone 125 mg was added to prevent slow-onset reactions, and the patient was admitted for further observation. His vital status was stable and he was discharged the next day. His ophthalmic condition recovered without topical or oral antibiotics.

**Figure 1:**
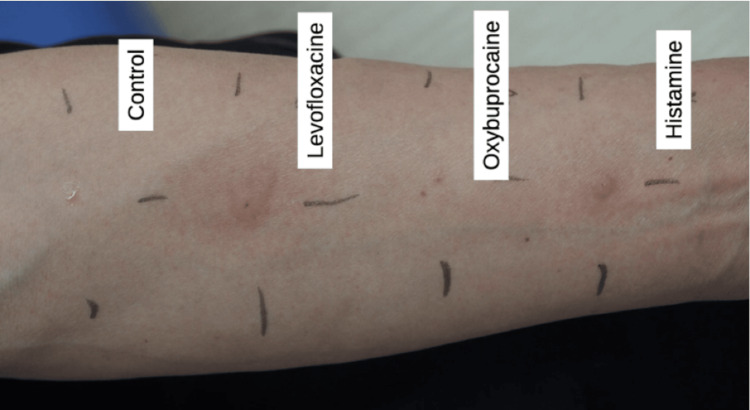
Prick test of eye drops From the left: control, levofloxacin, oxybuprocaine, positive control. Levofloxacin showed wheals of 19x13 mm, which was over twice the diameter of the 6.5x6.5 mm histamine chlorohydrate solution

Based on the clinical course, oxybuprocaine and levofloxacin eye drops were suspected to have caused anaphylaxis. Two weeks after the episode, prick testing of oxybuprocaine 0.04% and levofloxacin 1.5% eye drops was performed with negative/positive controls. Results for levofloxacin eye drops were positive (4+) (19x13 mm wheals), but results for oxybuprocaine were negative (-) within 15 minutes after application (Figure 1).

Levofloxacin eye drops consist of levofloxacin, glycerin, and a pH adjuster (hydrochloric acid and/or sodium hydroxide). The patient's IgE-mediated anaphylaxis due to levofloxacin was strongly suspected, and an additional skin test for pure levofloxacin reagent was considered. However, given the severity of the clinical symptoms and strong skin reactions, it was abandoned. He was given instruction to avoid any form of levofloxacin and ofloxacin. The patient had no similar episode until our telephone surveillance one year later.

## Discussion

We experienced a case where shock occurred immediately after the instillation of levofloxacin. In our case, there was no significant tracheal stenosis or eyelid edema, so the possibility of a vagal reaction must be excluded. This possibility is excluded by the persistence of hypotension and delayed tremor, as well as the absence of a history of similar symptoms, including the same treatment five years earlier. Our case is considered anaphylactic shock due to levofloxacin eye drops.

The levofloxacin eye drops were marketed in Japan in 2000 for the treatment of ocular infections. In contrast to the relatively high frequency of immediate hypersensitivity when administered orally or intravenously [3], levofloxacin eye drops have been used safely so far.

We found little literature on the occurrence of anaphylactic shock with these eye drops, and information regarding hypersensitivity to levofloxacin and its racemate form, ofloxacin eye drops, is provided by the manufacturer in the supplement information booklets [4,5]. One case of anaphylactic shock followed by toxicoderma and two cases of shock-like symptoms due to ofloxacin were described. One case of anaphylaxis and one case of shock-like reaction due to levofloxacin were described. None were confirmed by prick tests or drug provocation tests.

As far as we investigated, only one case of acute hypersensitivity to levofloxacin eye drops has been reported in the literature. The case was contact urticaria syndrome and confirmed by a prick test with 0.5% ophthalmic solution [6].

We speculate that the application of eye drops to a relatively deep defect after corneal foreign body removal may have caused the drug to diffuse into the eye and cause a strong hypersensitivity reaction.

We concluded that this case was a hypersensitivity to levofloxacin based on the (4+) (19x13 mm wheals) positive reaction seen on the prick test. However, the diagnostic value of skin tests in quinolone allergy remains controversial, mainly because quinolones have been reported to directly activate mast cells [7].

In our case, we decided to use 1.5% eye drops “as is” based on past reports. Saito and Nakada used 0.5% levofloxacin eye drops to obtain 6 mm wheal [6], and a case report investigating the cross-reactivity of ciprofloxacin and levofloxacin reported a negative prick test reaction to 5 mg/ml (0.5%) levofloxacin in a ciprofloxacin-sensitive patient [8]. We thought 1.5% eye drops have enough sensitivity and specificity.

However, levofloxacin has been reported to induce concentration-dependent histamine secretion from 300 μg/ml in rat peritoneal mast cells in vitro [7]. Skin prick test concentrations of 0.025-5 mg/ml are indicated in the literature [2], so a prick test with 1.5% (15 mg/dl) eye drops may increase the possibility of false positives. Since the optimal concentration of levofloxacin for prick testing has not yet been determined, it is preferable to perform a prick test using a dilution series to determine hypersensitivity to this drug.

## Conclusions

Our case is considered the first case of anaphylactic shock due to levofloxacin eye drops confirmed by a prick test. Levofloxacin eye drops carry a potential risk of severe hypersensitivity reactions, but a prick test may be helpful in diagnosis.
